# Functional characterization of the cannabinoid receptors 1 and 2 in zebrafish larvae using behavioral analysis

**DOI:** 10.1007/s00213-019-05193-4

**Published:** 2019-02-28

**Authors:** Floris J. Luchtenburg, Marcel J. M. Schaaf, Michael K. Richardson

**Affiliations:** Institute of Biology, Leiden University, Sylviusweg 72, 2333 BE Leiden, The Netherlands

**Keywords:** Zebrafish, Cannabinoids, Cannabinoid receptor 1, Cannabinoid receptor 2, Behavior, Visual motor response test

## Abstract

**Rationale:**

The endocannabinoid system (ECS) comprises the cannabinoids anandamide and 2-arachidonoylglycerol and the cannabinoid receptors 1 and 2 (Cnr1 and Cnr2). The function of these receptors in relation to zebrafish larval behavior is poorly understood, even though the zebrafish larva has become a versatile animal model in biomedical research.

**Objectives:**

The objective of the present study is to characterize the function of Cnr1 and Cnr2 in relation to behavior in zebrafish.

**Methods:**

Behavioral analysis of zebrafish larvae was performed using a visual motor response (VMR) test, which allows locomotor activity to be determined under basal conditions and upon a dark challenge.

**Results:**

Treatment with the non-specific Cnr agonists WIN55,212-2 and CP55,940 resulted in a decrease in locomotion. This was observed for both basal and challenge-induced locomotion, although the potency for these two effects was different, which suggests different mechanisms of action. In addition, WIN55,212-2 increased the reaction time of the startle response after the dark challenge. Using the Cnr1 antagonist AM251 and a *cnr1*^−/−^ mutant line, it was shown that the effects were mediated by Cnr1 and not Cnr2. Interestingly, administration of the antagonist AM251 alone does not have an effect on locomotion, which indicates that endogenous cannabinoid activity does not affect locomotor activity of zebrafish larvae. Upon repeated dark challenges, the WIN55,212-2 effect on the locomotor activity decreased, probably due to desensitization of Cnr1.

**Conclusions:**

Taken together, these results show that Cnr1 activation by exogenous endocannabinoids modulates both basal and challenge-induced locomotor activity in zebrafish larvae and that these behavioral effects can be used as a readout to monitor the Cnr1 responsiveness in the zebrafish larva model system.

## Introduction

The endocannabinoid system (ECS) is a neuromodulatory system that consists of the cannabinoid receptors 1 and 2 (Cnr1 and Cnr2 respectively), the endogenous ligands anandamide and 2-arachidonoylglycerol (AEA and 2-AG respectively), and the metabolic enzymes involved in synthesis or degradation of those ligands. The Cnr1 is a presynaptic G protein–coupled receptor (GPCR), which upon activation inhibits adenylate cyclase and N- and P/Q-type Ca2^+^ channels, and activates K^+^ channels, leading to inhibition of neurotransmitter release. The Cnr1 can regulate synaptic neurotransmission of excitatory and inhibitory circuits throughout the central nervous system (CNS). As a result, the ECS is important in regulating aspects of brain function, including mood, anxiety, appetite, and memory consolidation, and the control of locomotor activity. Like Cnr1, Cnr2 is a GPCR and also mediates its action via inhibition of adenylate cyclases (Ibsen et al. 2017). It is most abundantly present on cells of the immune system and has anti-inflammatory effects (Cabral and Griffin-Thomas 2009). Atwood and Mackie (2010) suggested that it might be the more peripherally located cannabinoid receptor, because initial research on the Cnr2 did not show any expression in the CNS. However, recent data have shown both expression and functional effects of the Cnr2 in the brain (Atwood and Mackie 2010; Chen et al. 2017).

The psychoactive component of the cannabis plant (*Cannabis*, marijuana), Δ9-tetrahydrocannabinol (THC), has been known for many years to affect animal behavior, such as aggressiveness, memory, dominance, and locomotion (Grunfeld and Edery 1969). The role of the ECS on locomotion led to an increased interest for cannabinoids as a potential (symptomatic) treatment against locomotor-related diseases, such as Parkinson’s disease, Huntington’s disease, or spasticity (Romero et al. 2002). After the discovery of Cnr1 in 1990 (Matsuda et al. 1990), it was shown in rodents that several agonists for this receptor have an inhibitory effect on locomotion (Anderson et al. 1996; Richter and Loscher 1994). However, there have sometimes been ambiguities in the behavioral data (Drews et al. 2005; McGregor et al. 1996; Polissidis et al. 2013), possibly due to differences among genetic strains of experimental animal, or differences in protocols such as the route of administration or dosage and exposure time.

In the present study, we have used zebrafish larvae to investigate the effects of Cnr1 and Cnr2 activation on locomotion. This model is a well-developed animal model for biomedical research and can be used as a complementary model to rodents (Ahmad et al. 2012; Kalueff et al. 2014; Khan et al. 2017; Stewart et al. 2014). Several features have made the zebrafish larval model increasingly popular. Zebrafish larvae can easily be obtained in large numbers, and their small size, rapid development, and optical transparency allow for phenotypic screening in relatively large numbers of replicates (Kimmel et al. 1995). In addition, the availability of tools for genetic manipulation and the availability of the entire genomic sequence enables genetic studies in this model (MacRae and Peterson 2015; Varshney et al. 2015).

Over the last decade, the ECS of zebrafish has been characterized and it was shown that it contains the same receptors, ligands, and metabolic enzymes as its mammalian equivalent (Krug and Clark 2015; McPartland et al. 2007). Interestingly, the metabolic enzyme Faah2 is absent in mice, but is conserved in both humans and zebrafish (Krug et al. 2018). In 2006, the expression of the *cnr1* gene was analyzed in zebrafish larvae and adults by in situ hybridization (Lam et al. 2006). This was followed by spatial analysis of *cnr2*, the gene responsible for encoding Cnr2 (Rodriguez-Martin et al. 2007), and developmental analysis of *daglα,* the gene encoding the metabolic enzyme Daglα (Watson et al. 2008). Oltrabella et al. 2017 recently presented an expression profile of zebrafish ECS genes during embryogenesis. Most of the investigated genes were stably transcribed after 48 h postfertilization (hpf), such as *cnr1*, *cnr2*, *mgll*, *dagl*, *faah*, *faah2*, and *napepld*.

Only a few functional studies have been done on the role of the ECS on behavior in zebrafish larvae. Chronic exposure to Cnr1 antagonist AM251 resulted in a lower hatching rate at 72 hpf and a dramatic decrease of motility at 96 hpf, while the developmental morphologic stages stayed the same (Migliarini and Carnevali 2009). Embryonic exposure to THC resulted in a reduced number of spontaneous muscle twitches while the embryos appeared morphologically normal (Thomas 1975).

Other subjects on the ECS in zebrafish larvae have been investigated as well, such as lipid metabolism (Nishio et al. 2012), leukocyte migration (Liu et al. 2013), and development (Akhtar et al. 2013; Migliarini and Carnevali 2009), and a number of studies have been performed on adult zebrafish (for a recent overview of work on the ECS in zebrafish, see Krug and Clark 2015).

Here, we aim to use zebrafish larvae to determine the role of Cnr1 and Cnr2 on locomotion. For this purpose, we have analyzed behavior of zebrafish larvae using a visual motor response (VMR) test, which includes both a bright phase and a dark phase or “challenge” of 4 min. Zebrafish larvae display escape and avoidance behavior in response to threatening tactile, acoustic, or visual stimuli (Colwill and Creton 2011). Because zebrafish larvae are scotophobic (averse to darkness) (Maximino et al. 2010; Steenbergen et al. 2011), the VMR assay allows for analyzing anxiety-like behavior such as hyperactivity and a startle response when the lights are turned off (Burgess and Granato 2007; Ellis et al. 2012; Peng et al. 2016), in addition to basal locomotion when the lights are on.

In order to determine the role of Cnr1 and Cnr2 in mediating the observed locomotor effects, we exposed the larvae to specific cannabinoid receptor agonists and antagonists and we utilized a *cnr1*^−/−^ mutant line (Liu et al. 2016). Studies done in other animal models showed that activation of Cnr1 affects motor behavior (Rodriguez de Fonseca et al. 1998; Wiley et al. 2014), whereas Cnr2 is generally considered to be psychoinactive (Fernandez-Ruiz et al. 2007). It can therefore be hypothesized that only modulation of Cnr1 affects locomotion, but it should be noted that receptor specificity may vary between species (Atwood and Mackie 2010). Our data show that activation of Cnr1 by exogenous cannabinoids results in a strong dose-dependent inhibition of both basal and dark challenge–induced locomotion in zebrafish larvae. Interestingly, inactivation of Cnr1 does not have an effect on locomotion, suggesting that endogenous cannabinoids are not involved in the regulation of locomotor activity at this stage of development.

## Materials and methods

### Embryo care

Fish were maintained and handled according to the guidelines on the ZFIN website (ZFIN, http://zfin.org). Fertilization was performed by natural spawning (group crossings), and eggs were initially raised in 10-cm Petri dishes containing 50 mL of 10% Hanks’ balanced salt solution (HBSS, for specifications see Ali et al. 2011), on a 14 h light to 10 h dark cycle at 28 °C. At 1 day postfertilization (dpf), the eggs were put individually in a 96-well plate (Costar 3599, Corning Inc., NY, USA) with 250 μL 10% HBSS. The larvae were left until 5 dpf. All analyses were performed at 5 dpf between 11:00 and 15:00. Tubingen (Tu) wild-type fish were used, as well as the cannabinoid receptor 1 mutant line *cnr1*^−/−^ (Liu et al. 2016), kindly provided by Prof. Wolfram Goessling of Harvard Medical School.

### Test compounds

The following compounds were used: WIN55,212-2, HU-910, and AM251 (Hoffmann-La Roche, Switzerland); CP55,940, (−)-nicotine (Sigma-Aldrich, MO, USA); JWH-133 (Tocris Bioscience, UK); and ethanol (98% purity; Boom, The Netherlands). All compounds were dissolved in 10% HBSS, and dimethylsulfoxide (DMSO) was used as a carrier (final concentration of 0.08% DMSO). Larvae treated with vehicle (0.08% DMSO in 10% HBSS) showed no difference in activity compared to the control group (10% HBSS). The applied concentrations were based on pilot experiments; lower concentrations were ineffective while higher concentrations were toxic. When the treatment consisted of exposure to 1 compound, 50 μL of this compound was added to a total volume of 300 μL. When the treatment consisted of exposure to 2 compounds, 25 μL of the first compound was added, 15 min later followed by addition of 25 μL of the second compound.

### Behavioral analysis

After addition of the compound(s), the 96-well plate was transferred to the recording apparatus (ZebraBox, Viewpoint S.A., France) and the recording started immediately after. The experimental recording consisted of three steps. First, larvae were acclimated to the behavioral setup with lights on for 4 min. This period was kept short since the Cnr1 is known to become rapidly desensitized upon prolonged activation (Hsieh et al. 1999). Second, a dark challenge of 4-min lights off was applied, which results in hyperactive behavior. Third, the larvae were left to recover for 30 min with the lights on. To investigate the effect of desensitization of the Cnr1, a different protocol was introduced. In this protocol, the 4 min lights on acclimatization phase was followed by 3 rounds of alternating 4 min lights off and 30 min lights on periods. Videos were recorded using FlyCapture software (Point Grey, Canada) at 24 frames per second and were analyzed using EthoVision 10 XT (Noldus, The Netherlands). Larvae that were dead at the beginning of the experiment were excluded from the analysis. The activity of each larva was assessed by determining the distance moved during 1-min periods and is presented as average velocity (mm/min). We defined the startle response as a movement with a minimum velocity of 15 mm/s during the first 5 s after the lights went off. Using these thresholds, we excluded non-startle behavior. This approach was validated by analyzing our videos for embryos with a C- or O-shaped body flexure (Burgess and Granato 2007; Eaton et al. 1977), which is a startle characteristic. Each experiment was performed three times, using a different clutch of eggs each time. Data shown are means of all larvae ± standard error of the mean (SEM).

### Statistics

The experimental data were analyzed with a one-way analysis of variance (ANOVA) with the concentration or compound as variable. A Dunnett’s post hoc test was performed to analyze multiple comparisons, and statistical significance was reported at *P* ≤ 0.05. All analyses were done and all graphs created with GraphPad Prism 7 (GraphPad Software Inc., CA, USA).

## Results

### The visual motor response (VMR) test

**Fig. 1:**
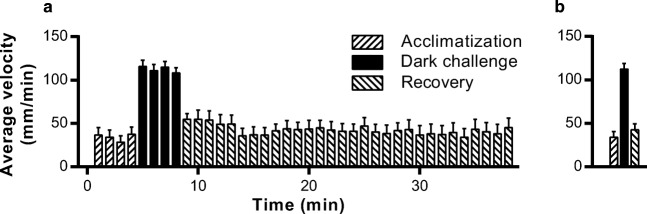
Behavior of zebrafish larvae assayed using the VMR test. **a** Average swimming velocities of vehicle-treated larvae per 1-min interval. In the first 4 min, the larvae acclimatize, with the lights on (“Acclimatization”). This period is followed by a 4-min dark challenge, which is associated with increased locomotor activity, reflecting anxiety-like behavior (“Dark challenge”). In the final phase, the fish are allowed to recover for 30 min with the lights on again (“Recovery”). **b** The average velocities from each phase were determined, and these average values are presented in a bar graph. This type of graph is used also in Figs. 2, 3, 4, 5, 6, 7 and 8. Data shown are means ± SEM

In the present study, a behavioral assay often referred to as the visual motor response (VMR) test (Emran et al. 2008) has been used to investigate the role of the ECS on swimming kinematics in zebrafish larvae. In this assay, the larvae are first allowed to acclimatize to the setup and then anxiety-like behavior is induced by turning off the light (Ellis et al. 2012; Peng et al. 2016). There is an increased swimming velocity during the dark period (Fig. 1a). When the lights are turned on again, the fish recover and locomotion rapidly returns to basal levels. The graph presented in Fig. 1b shows the average velocities during all three phases of the experiment, and in the following figures, data will be presented like this.

### Dual Cnr agonists, but not Cnr2 agonists, decrease locomotor activity

**Fig. 2:**
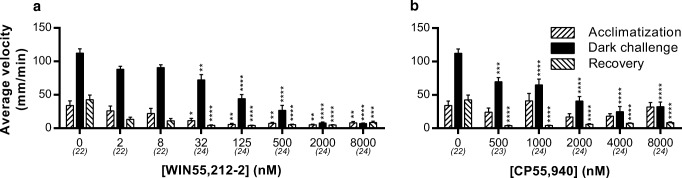
Effect of dual Cnr agonists on the average swimming velocity in the VMR test. **a** The effect of WIN55,212-2. This agonist causes a dose-dependent inhibition of swimming velocity in both the light and dark phases. **b** The effect of CP55,940. This agonist also inhibits locomotion in the light and in the dark phase in a dose-dependent way. Group sizes are reported in parentheses. Data shown are means ± SEM. Significant differences compared to the corresponding vehicle-treated control group are indicated.*P ≤ 0.05; **P ≤ 0.01; ***P ≤ 0.001; ****P ≤ 0.0001

The VMR test was used to investigate the behavioral effects of activation of Cnr1 and Cnr2. First, we administered the Cnr1 and 2 dual agonists WIN55,212-2 and CP55,940. These compounds are the most commonly applied and well-characterized Cnr agonists available. We found that WIN55,212-2 produced a dose-dependent reduction of locomotor activity in both the light and dark phases (Fig. 2a). In the concentration range 32–8000 nM, there was a significant, dose-dependent suppression of the average swimming velocity in both the light and dark phases compared to controls (vehicle only). Treatment with another dual Cnr agonist, CP55,940, also resulted in inhibitory effects on locomotion (at 500 nM and higher, Fig. 2b). The maximum inhibitory effect for the dark phase is reached at 2000 nM for WIN55,212-2. However, the maximum inhibitory effect for the acclimatization and recovery phase is reached at lower concentrations (125 nM and 32 nM, respectively).

**Fig. 3:**
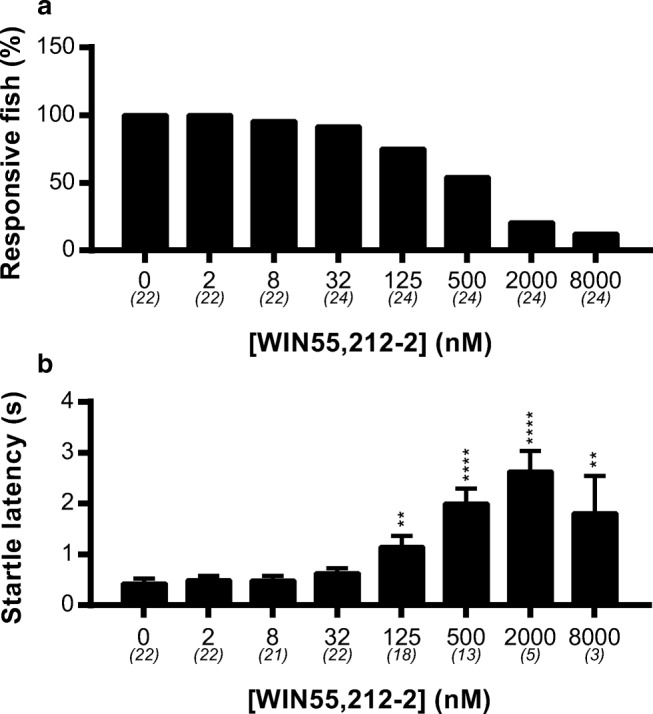
The effect of WIN55,212-2 on the startle response after a dark challenge. The behavior of the larvae during the first 5 s of the dark challenge was analyzed. **a** Percentage of larvae responding to the dark challenge by showing increased swimming velocity. From 125 nM and higher, a strong decrease of responsive fish can be noticed. **b** From the responsive fish, the reaction time was calculated. The latency was strongly reduced at concentrations of 125 nM and higher. Group sizes are reported in parentheses. Data shown are means ± SEM. Significant differences compared to the corresponding vehicle-treated control group are indicated. ***P ≤ 0.001; ****P ≤ 0.0001

The dark challenge induces a strong startle response, which is often observed as an immediate reaction to a threatening stimulus (Peng et al. 2016). We analyzed the effect of WIN55,212-2 on the startle response. The number of fish showing a startle response to the dark challenge showed a dose-dependent decrease after administration of WIN55,212-2 (Fig. 3a). Of 24 fish exposed to the concentrations of 2000 and 8000 nM, only 5 and 3 fish, respectively showed a startle response to the dark challenge. The startle latency (reaction time) of the responsive fish increased twofold at a concentration of 125 nM and fourfold at a concentration of 500 nM (Fig. 3b).

**Fig. 4:**
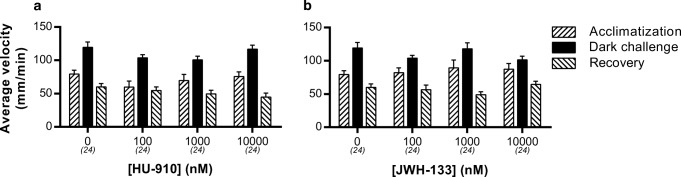
Effect of Cnr2 agonists on the average swimming velocity in the VMR test. **a** HU-910 and **b** JWH-133 have no effect on locomotion, in contrast to Cnr agonists WIN55,212-2 and CP55,940. Group sizes are reported in parentheses. Data shown are means ± SEM

**Fig. 5:**
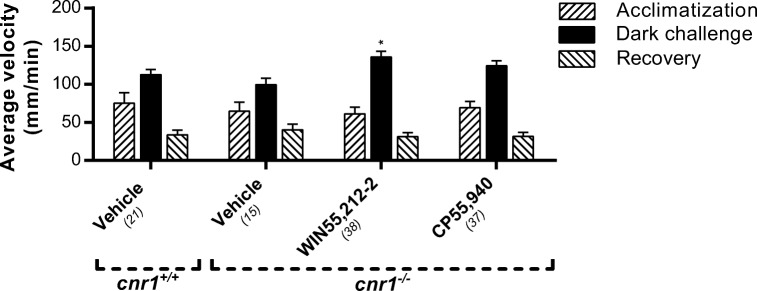
The effect of WIN55,212-2 and CP55,940 on locomotion in *cnr1*^−/−^ zebrafish larvae. The inhibitory effect of WIN55,212-2 (0.5 μM) and CP55,940 (2 μM) on locomotion in both the dark and the light phase, as observed in wild-type larvae (Fig. 2), was absent in the *cnr1*^−/−^ larvae. In fact, a slight increase in mobility during the dark phase was observed in the WIN55,212-2-treated larvae, as compared to the vehicle-treated larvae. No differences were found between the vehicle-treated *cnr1*^−/−^ and *cnr1*^+/+^ larvae. Group sizes are reported in parentheses. Data shown are means ± SEM. A significant difference compared to the corresponding vehicle-treated control group is seen. *P ≤ 0.05

To investigate whether the inhibiting effect of WIN55,212-2 and CP55,940 was Cnr1 or Cnr2 mediated, we applied the specific Cnr2 agonists HU-910 and JWH-133. Administration of these two compounds did not result in any effect on locomotion, either during the basal phase or dark-challenge phase (Fig. 4). To validate if this inhibiting effect on locomotion was thus Cnr1 mediated, we used a Cnr1 mutant line (Liu et al. 2016). In these *cnr1*^−/−^ larvae, we found no inhibitory effect of WIN55,212-2 or CP55,940 on the average swimming velocity in either the light or dark phases (Fig. 5). In fact, there was an opposite off-target effect: the velocity in the dark phase was increased by WIN55,212-2.

### The Cnr1 antagonist AM251 does not affect locomotor activity, but blocks the effect of WIN55,212-2

**Fig. 6:**
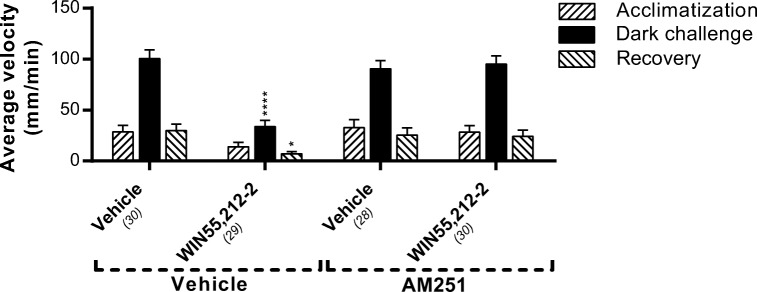
The effect of AM251 on locomotion of WIN55,212-2-treated zebrafish larvae. Administration of the Cnr1 antagonist AM251 (0.5 μM) showed no effect on swimming velocity but abolished the effect of WIN55,212-2 (125 nM) on locomotion. Group sizes are reported in parentheses. Data shown are means ± SEM. Significant differences compared to the corresponding phase of the vehicle/vehicle-treated control group are indicated. *P ≤ 0.05; ****P ≤ 0.0001

To investigate the effect of a pharmacological inhibition of Cnr1, we applied the Cnr1 antagonist AM251 in our assay. Treatment with AM251 (0.5 μM) did not affect locomotor activity in either the light or the dark phase, suggesting that endogenous Cnr1 agonists (AEA and 2-AG) do not affect locomotor behavior under these conditions. However, AM251 pre-treatment blocked the inhibitory effect of WIN55,212-2 (125 nM) treatment on locomotion. The antagonist abolished the WIN55,212-2 effects on the average velocity in both the light and the dark phase (Fig. 6).

### Ethanol and nicotine can increase locomotor activity in the presence of WIN55,212-2

**Fig. 7:**
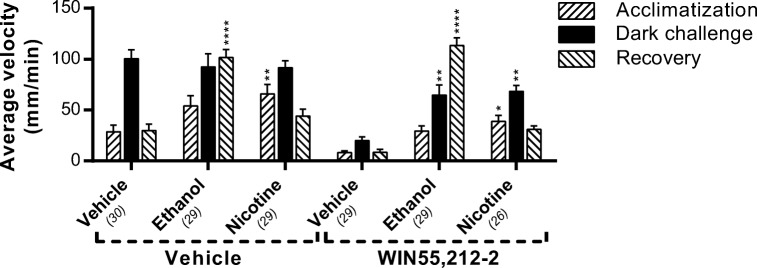
The effect of ethanol and nicotine on locomotion of WIN55,212-2-treated larvae. Administration of ethanol (1% *v*/*v*) and nicotine (10 μM) to WIN55,212-2-pre-treated (125 nM) larvae increases the locomotion in both the light and dark phases, indicating that the immobility induced by the Cnr1 agonist is not due to a physical limitation. Group sizes are reported in parentheses. Data shown are means ± SEM. Significant differences compared to the corresponding phase of the vehicle/vehicle or WIN55,212-2/vehicle-treated control group are indicated. *P ≤ 0.05; **P ≤ 0.01; ****P ≤ 0.0001

To study whether the inhibitory effect of WIN55,212-2 on locomotor activity is an effect of a decreased ability to move, we administered ethanol (1% *v*/*v*) 15 min after treatment with WIN55,212-2 (125 nM). Acute ethanol exposure is known to increase the locomotor activity of zebrafish larvae (Guo et al. 2015; MacPhail et al. 2009). In our assay, ethanol indeed increased the swimming velocity of the larvae. Interestingly, ethanol administration also increased the locomotor activity in the presence of WIN55,212-2 in both the light and the dark phase (Fig. 7).

A similar experiment was performed using nicotine, which has also been shown to increase the locomotor activity of zebrafish larvae as well (Petzold et al. 2009). Similarly to ethanol treatment, nicotine treatment (10 μM) increased the swimming velocity of the larvae. This treatment also increased the locomotor activity in the presence of WIN55,212-2 in both the light and dark phases.

### Desensitization of Cnr1

**Fig. 8:**
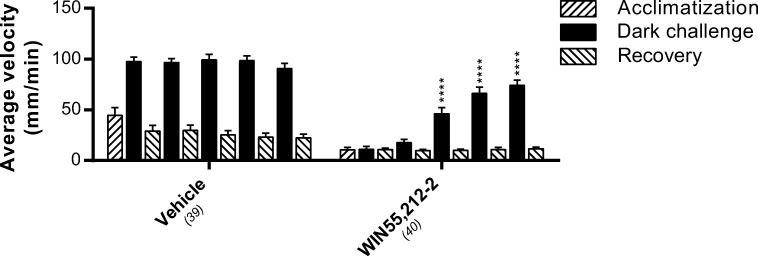
The effect of WIN55,212-2 on average swimming velocity upon repeated dark challenges. The response to repeated dark challenges did not significantly change in vehicle-treated larvae. WIN55,212-2 (2 μM) abolished the response to the first two dark challenges, but a reduced effect of this Cnr1 agonist was observed on the response to the third, fourth, and fifth dark challenges, indicating receptor desensitization. Group sizes are reported in parentheses. Data shown are means ± SEM. A significant difference compared to the first dark challenge is indicated. ****P ≤ 0.0001

G protein–coupled receptors (GPCRs) can become desensitized upon prolonged activation (Gainetdinov et al. 2004). Therefore, we tested the desensitization of the Cnr1 upon activation by WIN55,212-2 (2000 nM). Larvae were exposed to three subsequent dark challenges, separated by 30 min with lights on (Fig. 8). In the vehicle-treated larvae, all three dark challenges elicited a similar locomotor response and no differences between the locomotor activity of the light phases were detected. As observed before, WIN55,212-2 treatment abolished the behavioral response in the first dark challenge. However, the second dark challenge did elicit a response and this was increased in the third dark period (17.0 ± 4.8 and 48.4 ± 7.5 mm/min respectively), although it was still decreased compared to the vehicle-treated larvae. The decreased locomotor activity in the light phases did not change over time. These data demonstrate a desensitization of the Cnr1, which is reflected in a decreased inhibition of the behavioral response to a dark challenge, but not in a decreased inhibition of the mobility under light conditions.

## Discussion

In this study, we have functionally characterized the Cnrs in zebrafish larvae using a behavioral assay with pharmacological interventions. We have shown that the dual Cnr agonists WIN55,212-2 and CP55,940 have a pronounced dose-dependent inhibitory effect on zebrafish larval locomotion in the VMR test, both under basal conditions and after a dark challenge. These effects were not observed upon treatment with the Cnr2 agonists HU-910 and JWH-133. This shows that the inhibitory effects of WIN55,212-2 and CP55,940 on locomotion were Cnr1 mediated, which was also demonstrated using the Cnr1 antagonist AM251 and a *cnr1*^−/−^ mutant. Administration of the Cnr1 antagonist AM251 alone does not affect locomotion in our assay, which suggests that the endogenous cannabinoids are not active in regulating locomotor activity in the zebrafish larvae at the developmental stage studied here.

The maximum inhibitory effect of WIN55,212-2 is reached at lower concentrations in the light phase (125 nM) compared to the dark phase (2000 nM), which means these compounds show a higher potency in the light than in the dark (Fig. 2). This might be explained by the locomotor activity being higher in the dark than in the light. Complete inhibition of the locomotion may thus require more Cnr1 activation in the dark than in the light. Interestingly, WIN55,212-2 dose dependently decreases locomotion in the acclimatization phase, whereas CP55,940 does not. This discrepancy may be due to differences in the pharmacokinetics of these compounds, due to differences in for example skin adherence, absorption through the skin, and distribution through the body. Previously, it has been shown that WIN55,212-2 diffuses across human skin faster than CP55,940 (Valiveti et al. 2004).

To determine the specificity of the inhibitory effect of WIN55,212-2 in our zebrafish model, we applied the Cnr2 agonists HU-910 and JWH-133. These highly selective Cnr2 agonists do not inhibit locomotor activity, which is in line with the results obtained with the *cnr1*^−/−^ mutant in our study and data from other studies (Hanus et al. 1999; Malan et al. 2001). However, in other publications inhibition of locomotion after Cnr2 agonist exposure has been shown (Kruk-Slomka et al. 2017; Onaivi et al. 2008; Xi et al. 2011). Exposure of *cnr1*^−/−^ mutant larvae to WIN55,212-2 and CP55,940 did not result in any inhibitory effect on locomotion in these larvae. This indicates that the inhibitory effects are indeed Cnr1 mediated. In the same *cnr1*^−/−^ larvae, we found an increase in locomotor activity during the dark challenge (WIN55,212-2). This off-target effect may be due to developmental changes as a result of the Cnr1 deficiency, since it was not observed after co-administration with the Cnr1 specific antagonist AM251 (Fig. 6).

Different processes may be involved between the inhibition of locomotion upon Cnr1 activation in the dark phase and in the light phase. Zebrafish larvae are scotophobic (Maximino et al. 2010; Steenbergen et al. 2011) and show anxiety-like behaviors in the dark (Ellis et al. 2012; Peng et al. 2016). Because cannabinoids have anxiolytic properties (Korem et al. 2016; Morena et al. 2016; Patel et al. 2017), it could be that, next to the inhibition of motor functioning, a second, anxiety-related component is playing a role in the dark challenge. The locomotion is indeed lowered in the dark phase, but locomotion is also inhibited under basal circumstances (lights on). This suggests that locomotion itself is impaired due to the treatment, and with this test, we are thus not able to distinguish anxiety-related effects from locomotion-related effects in the dark phase. A more specific anxiety assay, such as the light-dark preference test (Steenbergen et al. 2011), should be used to study the potential anxiolytic properties of cannabinoids in zebrafish. Administration of WIN55,212-2 not only inhibits locomotion but also impairs the startle response. The number of larvae responding with a startle was reduced and the startle latency was increased. However, since the locomotion is reduced in the light phase as well, we cannot determine whether the inhibitory effect on the startle response is caused by an impaired motor system or if the startle reflex itself is affected. Using our images, we were not able to discriminate between different types of previously described startle responses of zebrafish. These responses include the C-bend that has been observed upon acoustic/vibrational stimuli and is mediated by Mauthner cells (Eaton et al. 1977), and the O-bend that has been described in response to a sudden decrement in light intensity (Burgess and Granato 2007). This latter response is independent of the Mauthner circuitry and considered to be primarily navigational. We suggest that the observed startle responses in our experiments most likely involve O-bends, since they are elicited by a dark stimulus (although it should be noted that the stimulus used in our study slightly differed from the dark flash demonstrated to elicit O-bends (Burgess and Granato 2007)).

Interestingly, AM251 alone does not alter the swimming kinematics, a finding consistent with the study of Akhtar et al. (2013), who found no effect of 0.5 mg/L (0.9 μM) AM251 on locomotion in 5 dpf zebrafish larvae. Higher concentrations (> 4 μM) resulted in toxic effects, which could explain the reduced locomotion found by Akhtar et al. at concentrations of 4 mg/L (7.2 μM) or higher. Our data indicate that, at the early larval stages of development, the endocannabinoid levels are insufficient to modulate locomotion, or that the system is not sensitive enough yet to be modulated by these endogenous levels, even though a complete ECS (including the metabolic enzymes and endogenous ligands) is present in the developing zebrafish larvae (Martella et al. 2016; Oltrabella et al. 2017). Studies done on rodents showed different outcomes upon modulating endogenous signaling, with some researchers reporting inhibition of locomotion (Cosenza et al. 2000; Long et al. 2009; Mallet et al. 2008), while others found no effect (Celorrio et al. 2016; Komaki et al. 2015).

When we looked at the effect of the Cnr1 agonists on locomotion upon prolonged exposure, we found that the inhibitory effect decreases in the dark phase, while it remains in the light phase. We think that this reduction in the dark phase can be attributed to a mechanism commonly referred to as desensitization, which is a well-known effect for GPCRs (Rajagopal and Shenoy 2018).

In a previous study, the effect of longer exposure (1–96 h) of cannabinoids on larval zebrafish locomotion was investigated (Akhtar et al. 2013). In that study, 1-h exposure to relatively high concentrations (1.1–3.4 μM for WIN55,212-2, and 6–48 μM for CP55,940) were used, which must have resulted in desensitization of the receptors, according to our results. Using a light and dark protocol, it was found that cannabinoids THC, WIN55,212-2, and CP55,940 cause hyperlocomotion in the dark and hypolocomotion in the basal light phase. The hypolocomotion under basal conditions is in line with our data, whereas the hyperlocomotion in the dark phase is opposite to our results. Different mechanisms of action could play a role here. The relatively high concentrations combined with a relatively long exposure time may result in desensitization of the receptors and potential off-target effects (Hajos and Freund 2002; Hudson et al. 2016). We found desensitization after exposure to 2000 nM WIN (Fig. 8, third dark challenge), but also for 500 nM (third dark challenge, data not shown) and even faster for 8000 nM (second dark challenge, data not shown). Furthermore, the way of administration of the compounds could affect the behavior. Akthar et al. replaced 175 μL of the 250 μL of swimming water, whereas we added 50 μL of compound resulting in a final volume of 300 μL. Finally, different strains of zebrafish were used, which may show different behavior. Recently, it was shown for example that the AB and TL strain differ in baseline HPI-axis activity, habituation to acoustic stimuli, and motor behavior (van den Bos et al. 2017). These differences could contribute to the apparent discrepancies between their study and ours.

The cannabinoid-induced effect on locomotion is often associated with the modulating function of the ECS on dopamine transmission (Fernandez-Ruiz et al. 2010). Interestingly, Lam et al. (2006) reported co-localization of the *cnr1* mRNA and tyrosine-hydroxylase, the rate-limiting enzyme for dopamine synthesis, in the caudal region of the zebrafish hypothalamus. The authors suggest that this particular region may be involved in regulating locomotion. Another study found that dopamine receptors do indeed have a pronounced effect on locomotor development and motor activity in zebrafish larvae, although this was not related to any specific brain region (Lambert et al. 2012). In mice, it was shown that dopamine receptor 1 (D1R) agonist quinelorane and dopamine receptor 2 (D2R) agonist 6-Br-APB were both able to attenuate motor dysfunction caused by Cnr agonist levonantradol (Meschler et al. 2000). In rats, the Cnr1 antagonist rimonabant blocked the cataleptic effect of CP55,940 but was not able to block the catalepsy elicited by D1R and D2R antagonists (SCH 23390 and raclopride respectively) (Anderson et al. 1996). Interestingly, CP55,940 did potentiate the catalepsy induced by the D1R and D2R antagonists. This suggests that the ECS plays a role upstream of the dopamine receptors and may be able to modulate the endogenous dopamine tone, which is an interesting subject for future study.

Since at high concentrations of dual Cnr agonist WIN55,212-2 the fish in our study were completely immobile, we hypothesized that they were unable to swim. Therefore we tried to induce recovery of the fish by administering ethanol or nicotine. It has been shown previously that both ethanol (1%) and nicotine (10 μM) strongly induce locomotor activity (MacPhail et al. 2009; Petzold et al. 2009). Our results confirm that both ethanol and nicotine induce hyperlocomotion (in the recovery phase and acclimatization phase respectively), but do not in the dark phase. The locomotion in the dark phase may have reached its ceiling level and therefore cannot go any higher. The delayed response to ethanol compared with nicotine can probably be explained by a slower uptake rate of ethanol. Administration of either ethanol or nicotine increased locomotor activity even in the presence of WIN55,212-2, which shows that the ECS does not limit physical ability to swim and does not directly affect the motor neurons of the somatic nervous system. Since the locomotion-modulating effects of ethanol and nicotine are regulated by altering dopaminergic signaling (Arias et al. 2010; King et al. 2004), it is reasonable to assume that ethanol and nicotine overrule the effect of the ECS on the dopamine receptor. This suggests that the inhibiting effect of the ECS on locomotion is solely mediated by the dopamine receptor and is not caused by a direct effect on motor neurons.

In conclusion, we have shown that activation of Cnr1 in zebrafish larvae suppresses locomotion both in basal conditions and during a dark challenge. As a result, this study provides an assay which can be used to determine the sensitivity of the Cnr1 in vivo, using its behavioral effects as a readout. The activity of endogenous ligands for the Cnr1 does not affect the outcome of our assay, which makes it very suitable for studying the effects of exogenous manipulation. Therefore, this assay can be used as a tool for genetic and chemical screening to unravel novel pathways involved in the modulation of Cnr1 signaling and the link between Cnr1 activity, dopamine signaling, and locomotion.
